# Association of plasma proteomics with mortality in individuals with and without type 2 diabetes: Results from two population-based KORA cohort studies

**DOI:** 10.1186/s12916-024-03636-0

**Published:** 2024-09-27

**Authors:** Hong Luo, Agnese Petrera, Stefanie M. Hauck, Wolfgang Rathmann, Christian Herder, Christian Gieger, Annika Hoyer, Annette Peters, Barbara Thorand

**Affiliations:** 1 Institute of Epidemiology, Helmholtz Zentrum München, German Research Center for Environmental Health (GmbH), Neuherberg, Germany; 2 Institute for Medical Information Processing, Biometry and Epidemiology (IBE), Faculty of Medicine, Pettenkofer School of Public Health, LMU Munich, Munich, Germany; 3 Metabolomics and Proteomics Core, Helmholtz Zentrum München, German Research Center for Environmental Health (GmbH), Neuherberg, Germany; 4 German Center for Diabetes Research (DZD), Partner München-Neuherberg, Neuherberg, Germany; 5 Institute for Biometrics and Epidemiology, German Diabetes Center, Leibniz Center for Diabetes Research at Heinrich Heine University, Düsseldorf, Germany; 6 German Center for Diabetes Research (DZD), Partner Düsseldorf, Neuherberg, Germany; 7 Institute for Clinical Diabetology, German Diabetes Center, Leibniz Center for Diabetes Research at Heinrich Heine University, Düsseldorf, Germany; 8 Department of Endocrinology and Diabetology, Medical Faculty and University Hospital Düsseldorf, Heinrich Heine University, Düsseldorf, Germany; 9 Research Unit of Molecular Epidemiology, Helmholtz Zentrum München, German Research Center for Environmental Health (GmbH), Neuherberg, Germany; 10 Biostatistics and Medical Biometry, Medical School OWL, Bielefeld University, Bielefeld, Germany

**Keywords:** Proteomics, All-cause mortality, Type 2 diabetes, Cardiovascular mortality, Cancer-related mortality, Other-cause mortality, Cohort study

## Abstract

**Background:**

Protein biomarkers may contribute to the identification of vulnerable subgroups for premature mortality. This study aimed to investigate the association of plasma proteins with all-cause and cause-specific mortality among individuals with and without baseline type 2 diabetes (T2D) and evaluate their impact on the prediction of all-cause mortality in two prospective Cooperative Health Research in the Region of Augsburg (KORA) studies.

**Methods:**

The discovery cohort comprised 1545 participants (median follow-up 15.6 years; 244 with T2D: 116 total, 62 cardiovascular, 31 cancer-related and 23 other-cause deaths; 1301 without T2D: 321 total, 114 cardiovascular, 120 cancer-related and 87 other-cause deaths). The validation cohort comprised 1031 participants (median follow-up 6.9 years; 203 with T2D: 76 total, 45 cardiovascular, 19 cancer-related and 12 other-cause deaths; 828 without T2D: 169 total, 74 cardiovascular, 39 cancer-related and 56 other-cause deaths). We used Cox regression to examine associations of 233 plasma proteins with all-cause and cause-specific mortality and Lasso regression to construct prediction models for all-cause mortality stratifying by baseline T2D. C-index, category-free net reclassification index (cfNRI), and integrated discrimination improvement (IDI) were conducted to evaluate the predictive performance of built prediction models.

**Results:**

Thirty-five and 62 proteins, with 29 overlapping, were positively associated with all-cause mortality in the group with and without T2D, respectively. Out of these, in the group with T2D, 35, eight, and 26 were positively associated with cardiovascular, cancer-related, and other-cause mortality, while in the group without T2D, 55, 41, and 47 were positively associated with respective cause-specific outcomes in the pooled analysis of both cohorts. Regulation of insulin-like growth factor (IGF) transport and uptake by IGF-binding proteins emerged as a unique pathway enriched for all-cause and cardiovascular mortality in individuals with T2D. The combined model containing the selected proteins (five and 12 proteins, with four overlapping, in the group with and without T2D, respectively) and clinical risk factors improved the prediction of all-cause mortality by C-index, cfNRI, and IDI.

**Conclusions:**

This study uncovered shared and unique mortality-related proteins in persons with and without T2D and emphasized the role of proteins in improving the prediction of mortality in different T2D subgroups.

**Supplementary Information:**

The online version contains supplementary material available at 10.1186/s12916-024-03636-0.

## Background

Mortality rate is a powerful and accurate indicator of the overall health of a population. Notably, individuals diagnosed with type 2 diabetes (T2D) face an elevated risk of premature mortality, not only in terms of all-cause mortality but also concerning various causes of death, especially cardiovascular disease (CVD) and cancer, with risks being 1.27–4.26 times higher than for those without T2D [1, 2]. Hence, it is crucial to understand the underlying mechanisms affecting mortality across different T2D status and to implement effective preventive strategies tailored to these specific groups.

Circulating biomarkers have the potential to elucidate biological pathways contributing to disease, holding promise for future pathway‐specific therapies and personalized treatment approaches. High-throughput proteomics, potent technologies for biomarker discovery [3], has been employed in prior studies exploring the associations [4–18] and predictive performance [4, 8–13, 15, 16] with all-cause and cause-specific mortality, primarily focused on cardiovascular mortality [7, 8, 13–16]. Of note, these studies mainly focused on the general population [4–8, 18], patients with CVD [9–15] or renal diseases [16], leaving a conspicuous research gap where comprehensive investigations in individuals with and without baseline T2D are lacking. Given the reported associations and potential causal effect between T2D and protein levels [19, 20], it is likely that T2D might influence protein–mortality associations.

To address this gap, our study is the first to assess the association of plasma proteomics with all-cause and cause-specific mortality in individuals with and without T2D. Subsequently, we constructed protein-enriched models stratified by baseline T2D status, evaluating the extent to which these protein biomarkers enhance the prediction of all-cause mortality beyond traditional risk factors.

## Methods

### Study population

The present analysis focused on two population-based Cooperative Health Research in the Region of Augsburg (KORA) cohorts for discovery and validation. The inclusion and exclusion criteria are illustrated in Additional file 1: Figure S1.

### KORA S4 – Discovery sample

The KORA S4 study enrolled 4261 participants from 1999 to 2001 [21]. The KORA S4 discovery sample used in the present analysis was restricted to those aged 55–74 with available proteomics data (*n* = 1653). Following the exclusion of participants with missing proteomics data, missing covariables, those lost to follow-up, non-T2D cases, and those with unclear diabetes status, this study comprised 1545 participants followed for a median of 15.6 years (244 participants with T2D and 1301 participants without T2D). Prevalent T2D included individuals with self-reported and subsequently validated T2D and with newly diagnosed T2D based on an oral glucose tolerance test (OGTT) using World Health Organization criteria [22] or baseline HbA1c ≥ 6.5%. Self-reported diabetes diagnoses were validated by contacting the treating physicians or reviewing medical charts; only participants without confirmed diabetes underwent an OGTT [23].

### KORA-Age1 – Validation sample

The KORA-Age1 study included 9197 participants from four cross-sectional Monitoring of Trends and Determinants in Cardiovascular Disease (MONICA) / KORA surveys (Survey S1 in 1984/85, Survey S2 in 1989/90, Survey S3 in 1994/95, and Survey S4 in 1999/2001) born before 1943 [24]. The KORA-Age1 validation sample for the present analysis was restricted to those who participated in an onsite baseline examination in 2009 (*n* = 1079, aged 65–93 years). After exclusions following the discovery sample criteria, 1031 participants followed for a median of 6.9 years remained for analysis (203 participants with T2D and 828 without T2D). Due to the lack of OGTT, prevalent T2D was defined based on self-report (with subsequent validation) and baseline HbA1c ≥ 6.5% only. Notably, 231 participants from KORA-Age1 overlapped with the KORA S4 discovery sample because these participants fell into the studied age range for KORA-Age1. Since these participants were examined at two different time points in the two studies, we included them in the primary analyses of both studies and subsequently excluded them in a sensitivity analysis of the KORA-Age1 study.

### Measurement of protein biomarkers

Plasma concentrations of 276 proteins were measured using the CVD-II, CVD-III, and Inflammation panels from Olink® (Uppsala, Sweden) based on proximity extension assay (PEA) technology in KORA S4 and KORA-Age1. Log2-normalized protein expression values were provided [25], and these were standardized by division by their respective standard deviation within the complete dataset, before applying exclusions. The proteomics data underwent consistent quality control criteria, including exclusions for proteins with over 25% of values below the limit of detection (LOD), and with missing values. If a protein was included in two panels, the duplicate with fewer values below LOD and a lower inter-assay coefficient of variation was retained. For the pooled sample of KORA S4 and KORA-Age1, protein values of KORA-Age1 were adjusted using bridging factors from duplicate KORA S4 measurements run together with the KORA-Age1 samples. In total, 233 unique proteins passed quality control in KORA S4 [26] and 90 proteins with statistically significant associations with all-cause mortality were carried forward to KORA-Age1 for validation.

Additionally, in KORA S4, five of the validated protein biomarkers (interleukin-1 receptor antagonist protein [IL-1RA], IL-6, insulin-like growth factor-binding protein [IGFBP] 2, IL-8, and N-terminal prohormone brain natriuretic peptide [NT-proBNP]) were additionally measured in serum using sandwich enzyme-linked immunosorbent assay (ELISA) or electrochemiluminescence immunoassay (ECLIA) (IL-1RA: Quantikine ELISA human Il-1ra Kit (R&D Systems, Wiesbaden, Germany) [27]; IL-6: PeliKine Compact human IL-6 ELISA Kit (CLB, Amsterdam, the Netherlands) [28]; IGFBP 2: Human IGFBP2 Quantikine ELISA Kit (R&D Systems, Wiesbaden, Germany) [27]; IL-8: ELISA from Sanquin [Amsterdam, the Netherlands] [29]; NT-proBNP: ECLIA [Roche Diagnostics, Mannheim, Germany] [30]).

### Measurement of all-cause and cause-specific mortality

Participants from the KORA S4 and KORA-Age1 cohorts were followed for all-cause and cause-specific (cardiovascular, cancer-related and other-cause) mortality until November 2016, using death certificates coded according to the International Classification of Diseases (ICD) 9th Revision. Cardiovascular mortality includes diseases of the circulatory system (codes: 390–459) and sudden death with unknown causes (code: 798). Cancer-related mortality consists of neoplasms (codes: 140–208). Other-cause mortality consists of the remaining causes of death, for example, pneumonia (code: 486), chronic bronchitis (code: 491) and dementias (code: 290).

### Covariates

Standard physical and medical examinations were conducted at KORA S4 and KORA-Age1 [24, 31], encompassing questions on age, sex, smoking habits, education, alcohol consumption, physical activity, and medical history. Smoking status was classified as either current smoker or non-smoker. Educational attainment was recorded as completed years of schooling. Alcohol intake was divided into three categories: no consumption (0 g/day), moderate consumption (men: 0.1–39.9 g/day, women: 0.1–19.9 g/day), and high consumption (men: ≥ 40 g/day, women: ≥ 20 g/day), based on self-reported consumption of beer, wine, and liquor. Physical activity levels were determined as either active or inactive, considering the frequency and duration of weekly exercise throughout different seasons [31]. Medication usage was defined using Anatomical Therapeutic Chemical Classification System codes [32]. Total cholesterol and high-density lipoprotein cholesterol (HDL-cholesterol) were measured by enzymatic methods [32]. Body mass index (BMI) was calculated by dividing weight (kg) by height squared (m^2^). Systolic and diastolic blood pressure were taken on the right arm while seated, following the World Health Organization MONICA protocol [33].

### Statistical analysis

**Fig. 1:**
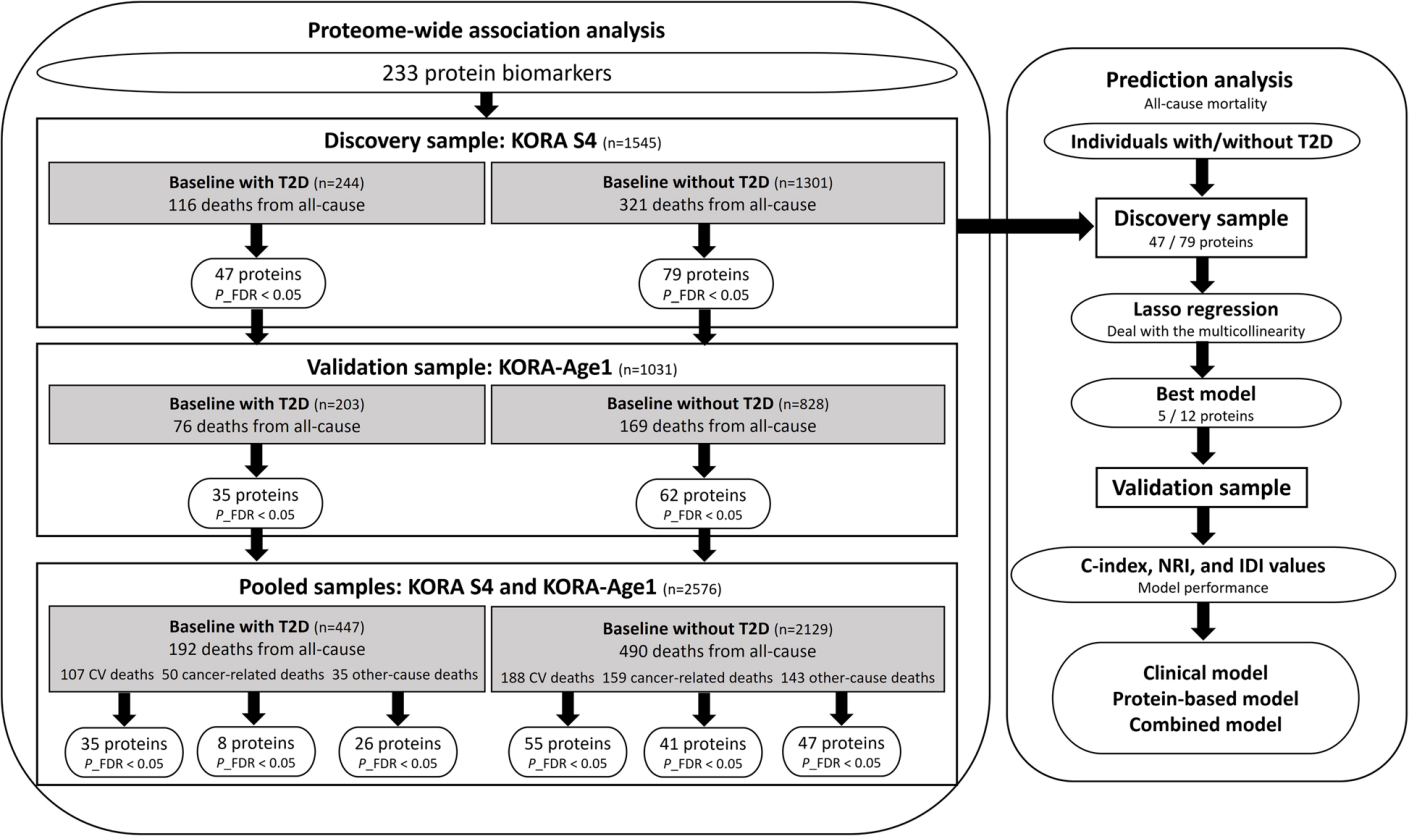
Analysis strategy of the present study. Abbreviations: C-index, concordance index; CV death, cardiovascular death; FDR, false discovery rate; IDI, integrated discrimination improvement; KORA, Cooperative Health Research in the Region of Augsburg; Lasso, least absolute shrinkage and selection operator; NRI, net reclassification index; T2D, type 2 diabetes

The analysis strategy of the study is shown in Fig. 1.

### Association analyses for all-cause and cause-specific mortality

Cox regression was used to determine associations between each protein and time-to-death among participants with and without T2D using the R package survival [34]. The assumption of proportional hazard was checked using the Schoenfeld residual test [35]. Model 1 included age and sex, while model 2 incorporated variables from the Framingham Risk Score [36] and the European Systematic COronary Risk Evaluation (SCORE) [37] / SCORE2 model [38] which are widely used for fatal and nonfatal CVD, encompassing age, sex, total cholesterol, HDL-cholesterol, systolic blood pressure, smoking status, and antihypertensive medication usage, along with additional relevant factors including BMI, education years, alcohol consumption, and physical activity. Proteins with significance in model 2 in KORA S4 were subsequently validated in KORA-Age1, considering a *P-*value < 0.05 after controlling for the Benjamini–Hochberg false discovery rate (FDR) as statistically significant.

Validated proteins of all-cause mortality were further examined for their associations with cause-specific mortality (cardiovascular, cancer-related, and other-cause mortality) in the pooled dataset of KORA S4 and KORA-Age1 to obtain more robust estimates.

### Pathway enrichment analysis

To elucidate potential connections and mechanisms of all-cause and cause-specific mortality, we annotated the validated proteins in the group with and without T2D using the STRING version 12.0 [39]. Subsequently, the network of identified proteins was constructed to identify pathways associated with respective all-cause or cause-specific mortality outcomes based on the Reactome pathway knowledgebase [40].

#### Prediction analysis for all-cause mortality

Three models were constructed for groups with and without baseline T2D, incorporating a protein-based model, a clinical model, and a combined model. All three models developed in KORA S4 were applied to KORA-Age1 for validation.

First, a protein-based model was constructed using a least absolute shrinkage and selection operator (Lasso) regression [41] to address multicollinearity. The 47 / 79 proteins that survived after FDR in the association analysis were retained for the Lasso regression. The penalization parameter λ was determined by five-fold cross-validation with Cox regression design with the R package glmnet [42]. The Lasso-selected proteins were included in the protein-based model and in the combined model. Second, a clinical model, corresponding to model 2 employed in the association analysis, was calculated. Finally, a combined model, that included the clinical risk parameters and the selected proteins, was derived.

While Harrel's concordance index (C‐index) has limitations in assessing model discrimination [43, 44], we augmented our evaluation with integrated discrimination improvement (IDI) [45] and category-free net reclassification improvement (cfNRI) [46]. R packages compareC [47] was used for the calculation of C-index and Hmisc [48] was used for cfNRI and IDI. Effect estimates were calculated as the arithmetic mean of these measures through five-fold cross-validation, with corresponding confidence intervals calculated from 200 bootstrap samples, using the R packages boot [49] and caret [50].

#### Sensitivity analysis

We excluded the 231 individuals who participated at two different time points in both KORA S4 and KORA-Age1 from the KORA-Age1 sample. Furthermore, we performed a sensitivity analysis using the Fine-Gray subdistribution hazard model to estimate protein–mortality associations for cardiovascular, cancer-related, and other-cause mortality over time in the presence of other causes of death specific to the corresponding mortality outcome as a competing risk. Correlations between the five protein biomarkers measured by other methods and their measurements by PEA technology were tested using Spearman's Rank correlation coefficient, and their associations with all-cause mortality were also evaluated.

The R version 4.3 (https://www.r-project.org/) was used for all analyses.

## Results

### Baseline characteristics of the study participants

Table 1 presents the characteristics of the study participants at baseline stratified by T2D status. All-cause and cause-specific mortality rates can be found in Additional file 1: Figure S1. In the KORA S4 study, over a median follow-up time of 15.6 years, 116 and 321 participants died (37.5 vs. 17.2 per 1000 person-years) in the group with and without T2D at baseline, respectively. Causes of death included 62 and 114 cardiovascular deaths (20.0 vs. 6.1 per 1000 person-years), 31 and 120 cancer-related deaths (10.0 vs. 6.4 per 1000 person-years), and 23 and 87 deaths from other causes (7.5 vs. 4.7 per 1000 person-years) in the group with and without T2D at baseline, respectively. In the KORA-Age1 study, over a median follow-up time of 6.9 years, 76 and 169 participants died (64.7 vs. 32.6 per 1000 person-years) in the group with and without T2D, respectively. Causes of death included 45 and 74 cardiovascular deaths (38.3 vs. 14.3 per 1000 person-years), 19 and 39 cancer-related deaths (16.2 vs. 7.5 per 1000 person-years), and 12 and 56 deaths from other causes (10.2 vs. 10.8 per 1000 person-years), respectively. Kaplan–Meier curves depicting the survival status of participants by baseline T2D status are shown in Additional file 1: Figure S2.

**Table 1:** Baseline characteristics of study population

Characteristics	Discovery sample—KORA S4				Validation sample—KORA-Age1			
	**With T2D (*n* = 244)**		**Without T2D (*n* = 1301)**		**With T2D (*n* = 203)**		**Without T2D (*n* = 828)**	
	**Death** **(*n* = 116)**	**No event** **(*n* = 128)**	**Death** **(*n* = 321)**	**No event** **(*n* = 980)**	**Death** **(*n* = 76)**	**No event** **(***n*** = 127)**	**Death** **(***n*** = 169)**	**No event** **(***n*** = 659)**
Median follow-up time (years)	15.6				6.9			
Age (years)	68 (64, 71)	63 (59, 67)	63 (68, 71)	62 (58, 67)	81 (76, 85)	76 (71, 80)	81 (77, 85)	74 (69, 79)
Male (%)	74 (63.8)	66 (51.6)	196 (61.1)	455 (46.4)	42 (55.3)	62 (48.8)	104 (61.5)	308 (46.7)
Systolic blood pressure (mmHg)	143.0 (128.4, 159.6)	144.8 (132.5, 155.0)	137.5 (124.5, 150.0)	131.8 (119.5, 145.0)	137.5 (123.5, 155.6)	139.0 (126.3, 150.3)	137.0 (122.0, 153.0)	137.0 (125.3, 149.3)
Diastolic blood pressure (mmHg)	80.8 (73.5, 89.1)	82.5 (77.0, 88.5)	80.0 (73.0, 87.0)	79.5 (73.0, 86.5)	72.0 (64.3, 79.8)	74.0 (66.0, 82.0)	72.5 (66.5, 82.0)	76.5 (70.0, 83.0)
Total cholesterol (mmol/l)	5.8 (5.3, 6.8)	6.0 (5.3, 6.7)	6.2 (5.5, 6.9)	6.4 (5.6, 7.0)	4.9 (4.2, 5.3)	5.2 (4.5, 6.0)	5.3 (4.4, 6.2)	5.5 (4.8, 6.2)
HDL-cholesterol (mmol/l)	1.2 (1.1, 1.6)	1.3 (1.0, 1.4)	1.5 (1.2, 1.8)	1.5 (1.2, 1.8)	1.2 (1.1, 1.5)	1.2 (1.1, 1.5)	1.4 (1.1, 1.7)	1.5 (1.2, 1.7)
BMI (kg/m^2^)	30.0 (27.7, 33.5)	30.2 (27.7, 33.5)	28.3 (25.9, 31.0)	27.5 (25.3, 30.2)	30.1 (26.9, 33.3)	30.4 (28.0, 34.3)	27.3 (24.8, 30.0)	27.5 (25.2, 30.2)
Education (years)	10 (8, 10)	10 (8, 11)	10 (10, 12)	10 (10, 12)	10 (8, 11)	10 (10, 11)	10 (10, 12)	10 (10, 12)
Physical activity (active, %)	27 (23.3)	44 (34.4)	107 (33.3)	470 (48.0)	26 (34.2)	70 (55.1)	64 (37.9)	395 (59.9)
Current smoker (%)	21 (18.1)	14 (10.9)	63 (19.6)	115 (11.7)	3 (3.9)	5 (3.9)	14 (8.3)	26 (3.9)
Alcohol consumption (%)								
None	39 (33.6)	49 (38.3)	84 (26.2)	260 (26.5)	35 (46.0)	54 (42.5)	60 (35.5)	219 (33.2)
Moderate	53 (45.7)	59 (46.1)	158 (49.2)	531 (54.2)	37 (48.7)	59 (46.5)	86 (50.9)	340 (51.6)
High	24 (20.7)	20 (15.6)	79 (24.6)	189 (19.3)	4 (5.3)	14 (11.0)	23 (13.6)	100 (15.2)
Medication use (%)								
Antihypertensive drugs	70 (60.3)	60 (46.9)	131 (40.8)	310 (31.6)	69 (90.8)	105 (82.7)	127 (75.1)	422 (64.0)
Statins	16 (13.8)	20 (15.6)	30 (9.3)	85 (8.7)	32 (42.1)	47 (37.0)	41 (24.3)	168 (25.5)
Lipid-lowering drugs	21 (18.1)	24 (18.8)	33 (10.3)	103 (10.5)	32 (42.1)	51 (40.2)	43 (25.4)	172 (26.1)
Fasting glucose (mmol/l) ^a^	7.1 (6.0, 7.8)	7.1 (6.3, 7.9)	5.5 (5.2, 6.0)	5.4 (5.1, 5.8)	-	-	-	-
2-h glucose (mmol/l) ^b^	11.8 (9.8, 13.6)	12.1 (10.2, 13.6)	6.4 (5.4, 7.6)	6.1 (5.1, 7.3)	-	-	-	-
Fasting insulin (pmol/l) ^c^	75.2 (44.6, 122.9)	86.0 (59.6, 127.8)	59.4 (41.4, 88.0)	58.5 (41.4, 82.8)	-	-	-	-
HbA1c (mmol/mol) ^d^	48.0 (42.0, 56.0)	45.5 (40.0, 53.0)	38.0 (36.0, 40.0)	38.0 (34.5, 40.0)	48.6 (42.8, 55.2)	47.5 (42.1, 50.4)	37.7 (34.4, 39.9)	36.6 (34.4, 38.8)
HbA1c (%) ^d^	6.5 (6.0, 7.3)	6.4 (5.8, 7.0)	5.6 (5.4, 5.8)	5.6 (5.3, 5.8)	6.6 (6.1, 7.2)	6.5 (6.0, 6.8)	5.6 (5.3, 5.8)	5.5 (5.3, 5.7)

Data are presented as median (25th, 75th percentile) for continuous variables and n (%) for categorical variables

*Abbreviations*: *BMI* body mass index; *HbA1c* haemoglobin A1c; *HDL* high‐density lipoprotein; *KORA* Cooperative Health Research in the Region of Augsburg; *T2D* type 2 diabetes

^a^Data were calculated in 138 participants with T2D (60 deaths vs. 78 no event) and 1208 participants without T2D (296 deaths vs. 912 no event) at KORA S4

^b^Data were calculated in 128 participants with T2D (55 deaths vs. 73 no event) and 1177 participants without T2D (289 deaths vs. 888 no event) at KORA S4

^c^Data were calculated in 142 participants with T2D (62 deaths vs. 80 no event) and 1176 participants without T2D (286 deaths vs. 890 no event) at KORA S4

^d^Data were calculated in 1299 participants without T2D (321 deaths vs. 978 no event) at KORA S4

### Association with all-cause and cause-specific mortality

In KORA S4, 47 and 79 proteins, including 36 overlapping proteins, showed significant associations with all-cause mortality in the group with and without T2D, respectively (Additional file 2: Table S1). Positive associations of 35 and 62 proteins, respectively, with 29 overlapping, were successfully validated in KORA-Age1 (Additional file 2: Table S2). The correlation between the validated proteins is shown in Additional file 1: Figure S3.

**Fig. 2:**
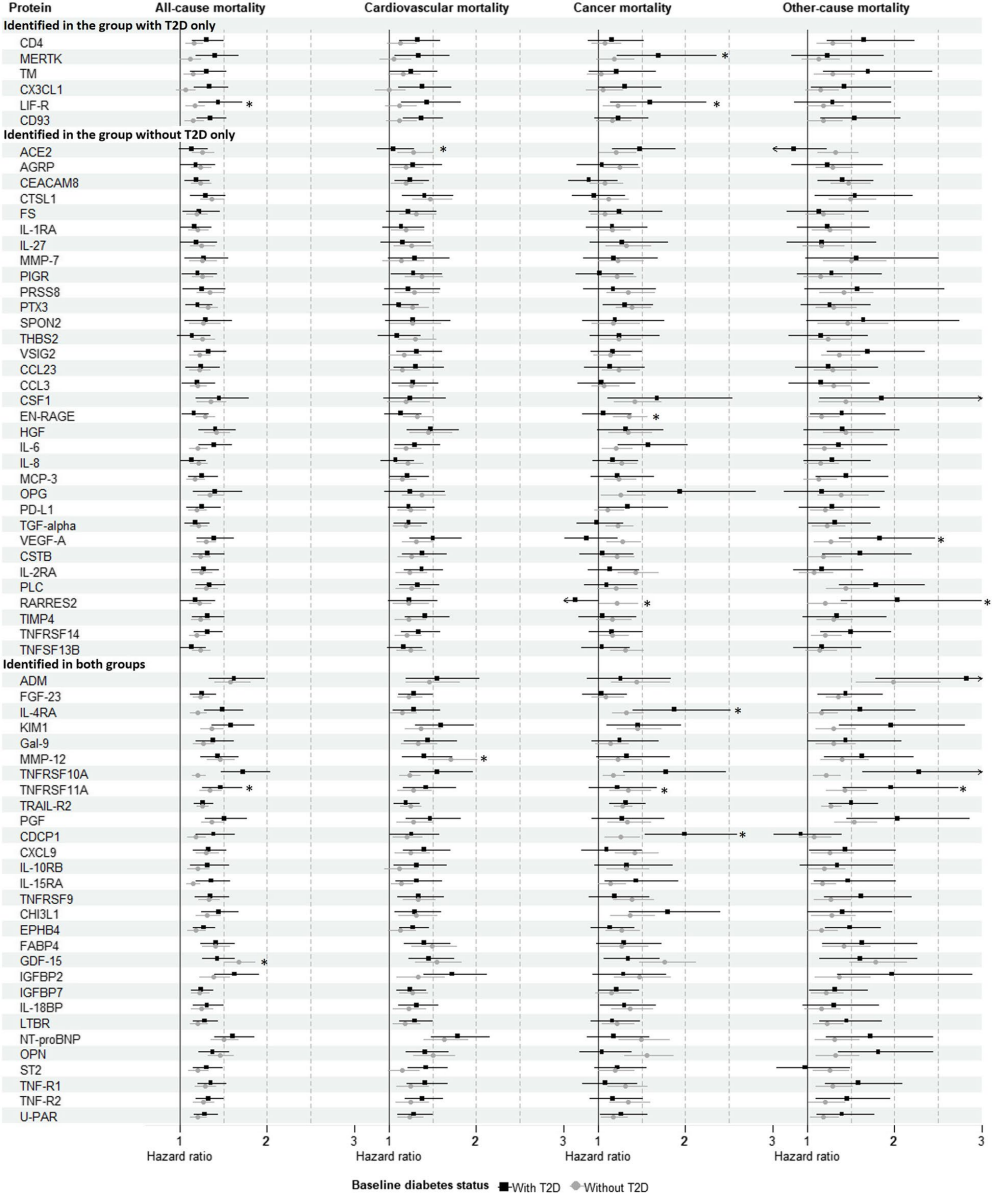
Association of validated 35 and 62 proteins for all-cause mortality in the groups with and without baseline type 2 diabetes (T2D), respectively, and their associations with all-cause and cause-specific mortality in the pooled sample. Hazard ratios have been calculated per 1 SD increase in normalized protein expression values on a log2 scale. Effect estimates and *P*-values were derived from Cox regression analysis adjusted for age, sex, total cholesterol, high‐density lipoprotein cholesterol, systolic blood pressure, antihypertensive medication use, current smoking, body mass index, education years, physical activity, and alcohol consumption (model 2). * indicates that the interaction term with T2D status at baseline was statistically significant (*P*-value < 0.05). The interaction effect of T2D status was examined by adding the term (protein × T2D status) to model 2 among all participants combined. Abbreviations: KORA, Cooperative Health Research in the Region of Augsburg; T2D, type 2 diabetes. Full names of the biomarkers can be found in Additional file 1: Table S1

Among the validated proteins of all-cause mortality, 35, eight, and 26 proteins were positively associated with cardiovascular, cancer-related, and other-cause mortality in participants with T2D, while 55, 41 and 47 proteins were positively associated with respective cause-specific outcomes in participants without T2D (Fig. 2 & in Additional file 2: Table S3-S4). Three (leukemia inhibitory factor receptor [LIF-R], tumor necrosis factor receptor superfamily member [TNFRSF] 10A, and growth/differentiation factor 15 [GDF-15]), two (angiotensin-converting enzyme 2 and matrix metalloproteinase-12 [MMP-12]), seven (tyrosine-protein kinase Mer [MERTK], LIF-R, protein S100-A12 [EN-RAGE], retinoic acid receptor responder protein 2 [RARRES2], interleukin-4 receptor subunit alpha [IL-4RA], CUB domain-containing protein 1, and TNFRSF10A), and three (RARRES2, TNFRSF10A, and vascular endothelial growth factor A) proteins demonstrated significant interaction effects with baseline T2D status (Additional file 2: Table S5) in the pooled dataset for all-cause, cardiovascular, cancer-related, and other-cause mortality, respectively.

After excluding overlapping KORA S4 participants from the KORA-Age1 sample, the identified significant associations of proteins with all-cause mortality remained significant in the pooled sample among both persons with and without T2D (Additional file 2: Table S6-S7). When considering competing risks, only three (IGFBP-2, NT-proBNP, and ST2) and four (IL-4RA, CUB domain-containing protein 1, TNF-related apoptosis-inducing ligand receptor 2 [TRAIL-R2], and chitinase-3-like protein 1 [CHI3L1]) proteins of the validated proteins in the group with T2D were significantly associated with cardiovascular and cancer-related mortality, respectively, while 15, 27 and four proteins remained significantly associated with the respective cause-specific outcomes in participants without T2D (Additional file 2: Table S8-S9).

The correlation coefficients of the five proteins measured by other methods and PEA technology ranged from 0.5250 to 0.8884 (Additional file 2: Table S10). Except for IL-8, the associations between IL-1RA, IL-6, and all-cause mortality in the group with T2D, as well as the associations between IGFBP-2, NT-proBNP, and all-cause mortality in both groups with and without T2D, were replicated.

### Mechanism network and related pathways of identified protein sets

**Fig. 3:**
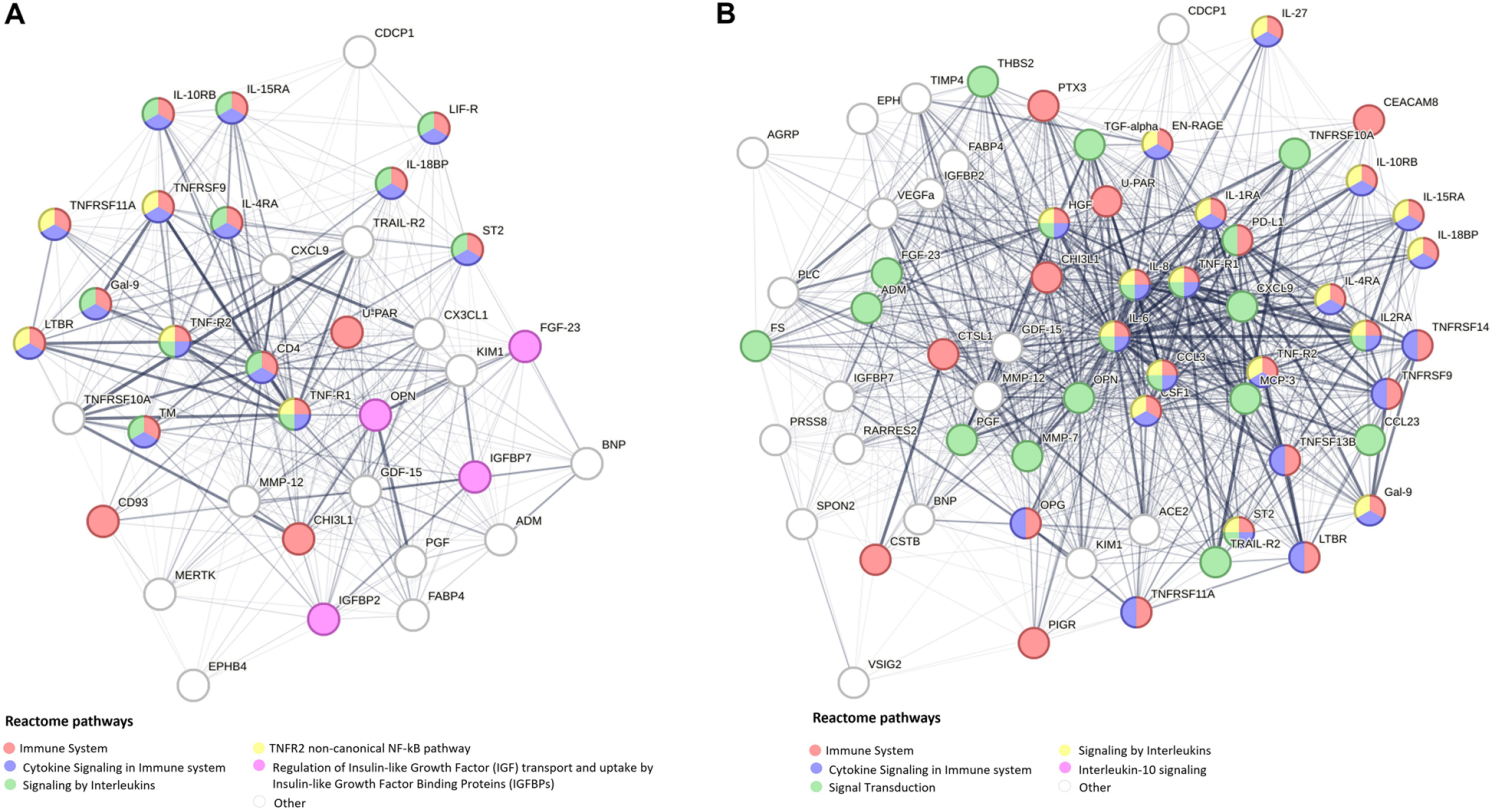
Protein–protein interaction networks of validated all-cause mortality-associated proteins among participants (**A**) with and (**B**) without type 2 diabetes at baseline. The edges between protein nodes represent the interaction score between the proteins from the STRING database considering all types of evidence. Only edges featuring interaction scores > .15 are displayed. The thickness of edges corresponds to the strength of data support. Node color signifies the Reactome pathway the protein is associated with. The five most enriched Reactome pathways are displayed. Abbreviations: Full names of the biomarkers can be found in Additional file 1: Table S1

The resulting protein–protein networks for all-cause mortality are presented in Fig. 3. Several pathways like the immune system and signaling by interleukins were overrepresented in both persons with and without T2D, while regulation of insulin-like growth factor (IGF) transport and uptake by IGFBPs was enriched exclusively in the group with T2D. Results were similar for cardiovascular mortality (Additional file 2: Table S11).

### Prediction of all-cause mortality

Five (NT-proBNP, GDF-15, TRAIL-R2, kidney injury molecule 1 [KIM1], and IGFBP-2) and 12 proteins (NT-proBNP, GDF-15, TRAIL-R2, KIM1, MMP-12, CHI3L1, prostasin, EN-RAGE, polymeric immunoglobulin receptor, fibroblast growth factor 23 [FGF-23], pentraxin-related protein PTX3, and IL-8), with four overlapping proteins, were selected to be included in the protein-based prediction model for all-cause mortality in those with and without T2D, respectively, using Lasso.

In the group with T2D, in KORA S4, the protein-based model displayed similar predictive performance as the clinical model with no significant improvements in any of the performance indicators, while the combined model showed significant improvements in ΔC-index, cfNRI, cfNRI_survivors_, and IDI, compared to the clinical model (Table 2 & Additional file 2: Figure S4). Results were similar in KORA-Age1. In the group without T2D, the model performance of all three models tended to be better (higher C-index) than in persons with T2D, but differences between the protein-based and combined models compared to the clinical model were similar to those in persons with T2D for both KORA S4 and KORA-Age1, with the combined model demonstrating the best predictive performance (Table 2 & Additional file 2: Figure S4).

**Table 2:** Predictive performance for all-cause mortality

Baseline status	KORA S4			KORA-Age1		
	**Clinical model**	**Protein-based model**	**Combined model**	**Clinical model**	**Protein-based model**	**Combined model**
**With T2D**^a^						
C‐index	0.672 [0.630; 0.739]	0.706 [0.662; 0.756]	0.722 [0.686; 0.780]	0.659 [0.622; 0.772]	0.717 [0.675; 0.802]	0.704 [0.681; 0.822]
ΔC‐index^c^	-	0.035 [-0.029; 0.092]	**0.050 [0.009; 0.090]**	-	0.058 [-0.032; 0.116]	**0.045 [0.005; 0.114]**
cfNRI	-	0.111 [-0.404; 0.433]	**0.481 [0.125; 0.664]**	-	0.112 [-0.446; 0.465]	**0.436 [0.191; 0.761]**
cfNRI_deaths_	-	0.062 [-0.194; 0.204]	0.011 [-0.113; 0.207]	-	0.087 [-0.205; 0.217]	0.096 [-0.125; 0.357]
cfNRI_survivors_	-	0.050 [-0.283; 0.279]	**0.470 [0.130; 0.569]**	-	0.025 [-0.324; 0.291]	**0.340 [0.222; 0.627]**
IDI	-	0.015 [-0.069; 0.107]	**0.089 [0.042; 0.149]**	-	0.057 [-0.068; 0.116]	**0.085 [0.032; 0.179]**
**Without T2D**^b^						
C‐index	0.715 [0.684; 0.747]	0.710 [0.686; 0.745]	0.741 [0.718; 0.780]	0.744 [0.724; 0.797]	0.779 [0.742; 0.816]	0.793 [0.759; 0.837]
ΔC‐index^c^	-	-0.005 [-0.034; 0.030]	**0.026 [0.016; 0.050]**		0.035 [-0.010; 0.072]	**0.049 [0.020; 0.077]**
cfNRI	-	-0.253 [-0.383; 0.006]	**0.217 [0.151; 0.432]**	-	0.091 [-0.167; 0.364]	**0.448 [0.237; 0.635]**
cfNRI_deaths_	-	-0.128 [-0.207; -0.007]	-0.046 [-0.107; 0.087]	-	0.075 [-0.093; 0.173]	0.053 [-0.089; 0.201]
cfNRI_survivors_	-	-0.125 [-0.205; 0.042]	**0.263 [0.185; 0.357]**	-	0.016 [-0.139; 0.222]	**0.395 [0.252; 0.472]**
IDI	-	-0.022 [-0.048; 0.013]	**0.030 [0.020; 0.056]**	-	0.009 [-0.032; 0.048]	**0.040 [0.023; 0.082]**

*Abbreviations*: *C‐index* concordance index; *cfNRI* category‐free net reclassification index; *IDI* independent discrimination improvement; *KORA* Cooperative Health Research in the Region of Augsburg; *T2D* type 2 diabetes

^a^The five selected proteins which were included in the protein-based model and the combined model in the group with type 2 diabetes are NT-proBNP, GDF-15, TRAIL-R2, KIM1, and IGFBP-2

^b^The 12 selected proteins which were included in the protein-based model and the combined model in the group without type 2 diabetes are NT-proBNP, GDF-15, TRAIL-R2, KIM1, MMP-12, CHI3L1, PRSS8, EN-RAGE, PIGR, FGF-23, PTX3, and IL-8

^c^The difference in concordance index (ΔC‐index) comparing the protein-based and the combined models with the clinical model was calculated according to the formula: ΔC‐index = C‐index _protein-based model / combined model_ - C‐index_clinical model_)

Bold indicates statistically significant value

In a sensitivity analysis excluding overlapping participants from KORA-Age1, except for the ΔC-index, which was not significantly improved in the T2D group, the prediction results demonstrated improved performance of the combined model compared to the clinical model in both T2D groups (Additional file 2: Table S12).

## Discussion

Using a discovery–validation approach, we examined the association of 233 protein abundance levels, measured by PEA-based technology, with all-cause and cause-specific mortality in individuals with and without T2D. In individuals with T2D, we identified 35 proteins that were positively associated with all-cause mortality, while 62 proteins with positive associations were identified in those without T2D. Interestingly, both sets of proteins shared common pathways, such as immune- and inflammatory-related pathways. However, regulation of IGF transport and IGFBPs emerged as a unique pathway in the T2D group, confirming that T2D-related pathways might contribute to premature mortality in persons with T2D. Of note, albeit the examined proteins were initially selected for their links to inflammation and CVD, the identified proteins linked to all-cause mortality demonstrated associations with all examined cause-specific outcomes, including cardiovascular, cancer-related, and other-cause mortality. While many of the identified protein–mortality associations have been previously reported [4–16, 51], our study identified four novel proteins associated with all-cause mortality. Furthermore, we showed that the addition of a limited number of proteins to prediction models based on clinical risk factors significantly improved the prediction of all-cause mortality for both persons with and without T2D.

### Novel protein candidates for all-cause mortality

A novel protein was defined as one lacking a significant association with all-cause mortality in previous epidemiological studies, such as those using proteomics measurements after controlling multiple testing. Consequently, we identified four novel proteins, including MERTK, IL-27, monocyte chemotactic protein 3 (MCP-3), and lymphotoxin-beta receptor (LTβR). MERTK, which was found specifically in the group with T2D, also exhibited a unique association with cancer mortality and demonstrated a significant interaction effect with T2D when examined in the total study group. MERTK is known to contribute to the oncogenesis of various human cancers [52, 53] and has been linked to atherosclerosis [54] and diabetes [55]. Although excessive circulating levels of MERTK have been associated with renal injury, especially in patients with T2D [56, 57], its role in the development of premature mortality in T2D remains undefined.

In the group without T2D, IL-27 was significantly associated with cardiovascular and cancer mortality in the present study. This pro-inflammatory cytokine has previously been associated with incident coronary heart disease [58] and various inflammatory diseases, including lung [59], sepsis [60], and hepatic injury [61]. Similarly, in the group without T2D, MCP-3 (also known as C–C motif chemokine 7 [CCL7]) showed a significant association with cancer-related mortality and a borderline significant association with cardiovascular mortality. Playing a crucial role in cell recruitment to inflammatory sites and diseases [62], dysregulation of MCP-3 has been linked to cardiac inflammation and impaired cardiac function [63]. Another novel biomarker observed in both groups with and without T2D was LTβR. In both T2D groups, LTβR was associated with cardiovascular and other-cause mortality, while among individuals without T2D only, it showed significant association with cancer-related mortality in this study. LTβR, a cell surface receptor and a member of the tumor necrosis factor receptor superfamily, is involved in various immunological and inflammatory pathways [64, 65], contributing to processes such as liver regeneration and lipid homeostasis [66, 67]. Notably, IL-27, MCP-3, and LTβR, in a population-based cohort study, were reported to have a positive but non-significant association with all-cause mortality after Bonferroni correction [4].

### Previous proteomics studies

Many of our validated all-cause mortality-associated proteins align with previous investigations using high-throughput technologies [4–13] (details see Additional file 2: Table S13). Our study successfully replicated 50 proteins among 1364 significant all-cause mortality-associated proteins identified using affinity-based proteomics (SOMAscan assay) in a population-based cohort (22,913 participants and 7061 deaths) [4]. Using the same type of assay, another prospective study (997 participants and 504 deaths) identified 193 proteins significantly associated with all-cause mortality, with 24 of them aligning with our findings [6]. Using PEA technology, in a prospective study (3918 participants with 974 deaths), four of their identified eight all-cause mortality-associated proteins showed consistent positive association with our findings [5]. In addition, a further cohort study (1713 participants and 590 deaths) explored seven diabetes-related proteins, revealing two of our validated proteins to be associated with both all-cause and cardiovascular mortality [7]. Moreover, another population-based study (3523 participants and 755 all-cause and 167 cardiovascular deaths) employed a modified ELISA technique, identifying 38 and 35 proteins to be associated with all-cause and cardiovascular mortality, respectively, with six proteins overlapping with our study [8].

Other proteomics population-based studies using mass spectrometry-based methods and nuclear magnetic resonance spectroscopy also explored associations with all-cause and cause-specific mortality [17, 18]. Further studies focused on all-cause mortality among patients with CVD [9–13]. Additionally, four proteomics studies explored associations with cardiovascular mortality among patients with CVD [13–15] and renal diseases [16].

Notably, proteins such as IL-6 [4, 6, 8, 10–15] identified in the group without T2D, FGF-23 [4, 8–15], TRAIL-R2 [4, 11–15], and GDF-15 [4–6, 8, 11, 12, 14, 15] identified in both groups with and without T2D in the present study, emerge as the most reported proteins for all-cause and cardiovascular mortality. These consistent findings across various studies underscore the robustness and clinical relevance of these proteins as potential markers for mortality risk.

However, our study distinguishes itself by specifically identifying biomarkers according to baseline T2D status, a novel approach compared to previous studies that assessed associations in population-based samples or in patients with CVD or renal diseases. Differences in targeted age groups, follow-up durations, and measurement techniques [25, 68] contribute to variations among studies.

### Prediction of all-cause mortality

Our study is the first to establish proteomics-enriched predictive models for all-cause mortality separately for those with and without prevalent T2D. The significant improvement in predictive performance by adding the selected proteins to a clinical prediction model, evident in both the discovery and validation samples, was reflected by a 6.8% and 6.6% increase in the C-index for the group with and without baseline T2D, respectively, in the validation sample. This enhancement underscores the clinical relevance and potential applicability of these protein-enriched models in clinical practice.

Notably, our finding highlighted proteins such as GDF-15 [4, 8, 11, 12], TRAIL-R2 [9, 11–13], KIM1 [9, 16], NT-proBNP [8], IGFBP-2 [8], and MMP-12 [9] as standout prognostic proteins for all-cause mortality, aligning partially with previous investigations in the general population or patients with CVD. For instance, Eiriksdottir et al. [4] developed prediction models with 98, 117, and 199 proteins for all-cause mortality at 2-, 5-, and 10-year intervals in a population-based sample, showcasing a 4.3%, 3.4%, and 2.4% improvement in C-index compared to age-sex-based protein models, respectively. In these models, GDF-15 emerged as the most powerful predictor. Similarly, in another population-based cohort with 14.3 years of follow-up, Ho et al. [8] constructed a 12-protein-based model that showed a 4.6% improvement in the C-index on top of clinical variables. Their constructed model included GDF-15, NT-proBNP, and IGFBP-2. Unterhuber et al. [9] established a 20-protein model in patients with CVD, demonstrating a 9.6–12.5% improvement in the C-index for 10-year all-cause mortality prediction compared to a baseline clinical model, which also included TRAIL-R2, MMP-12, and KIM1. Additionally, Skau et al. reported GDF-15 and TRAIL-R2 as potent predictors for 10-year all-cause mortality in patients with acute myocardial infarction [12] or peripheral arterial disease [11]. However, the practical application of these models in clinical settings requires cautious consideration due to variations in proteins, populations, and methodologies across studies.

### Strengths and limitations

We employed advanced targeted proteomics technology to investigate associations of a broad spectrum of proteins with mortality. A notable strength of our analysis strategy is the validation of the initially identified proteins in another study. Specifically examining protein–mortality associations by T2D status offered insights into the underlying mechanisms leading to mortality in individuals with and without T2D.

However, certain limitations of the present study need consideration. First, the PEA approach provided relative, rather than absolute, protein concentrations. Importantly, this difference did not affect the reported associations in this study, as evidenced by consistent results obtained with other measurements for a subset of proteins (Additional file 2: Table S10) [25]. Nonetheless, it has to be acknowledged that the availability of absolute protein measurements would facilitate the transfer of derived prediction models into clinical practice. Secondly, the limited number of deaths resulted in a relatively low power for detecting differences in cause-specific mortality. Therefore, we restricted analyses on cause-specific mortality to the proteins significantly related to all-cause mortality after validation and did not follow a stringent discovery–validation strategy for the identification of proteins related to cause-specific mortality outcomes based on all measured proteins. Furthermore, to enhance statistical power, we pooled samples from the discovery and validation cohorts to obtain more robust estimates for associations between proteins and cause-specific mortality and refrained from developing prediction models for cause-specific mortality outcomes. Additionally, although validation in KORA-Age1 reinforced the results for the validated proteins, we might lack replication for some proteins, especially if their impact was influenced by age, given that the KORA-Age1 participants were all older than 64 years. Moreover, it is noteworthy that there is some overlap between the participants of KORA-Age1 and KORA S4 albeit participants were examined at different time points. However, excluding these overlapping participants from our analyses did not lead to substantial changes. Finally, the shorter follow-up duration of KORA-Age1 compared to KORA S4 needs to be acknowledged as a limitation.

## Conclusions

In summary, our study identified common and distinct mortality-related proteins among individuals with and without baseline T2D, emphasizing the pivotal role of these proteins in mortality. The findings highlighted the significance of immune and inflammatory processes in both examined groups and the regulation of IGF transport and uptake by IGFBPs specifically in individuals with T2D. In addition, some variations in the most relevant proteins for improved mortality prediction were observed between those with and without T2D, underscoring the need to further explore disease-specific prediction models.

## Supplementary Information

Additional file 1:  Figure S1. Inclusion and exclusion criteria for the present study; Figure S2. Kaplan–Meier curves for all-cause mortality, cardiovascular mortality, cancer-related mortality, and other-cause mortality stratified by baseline type 2 diabetes status in KORA S4 and KORA-Age1; Figure S3. Correlation between the validated 35 and 62 protein biomarkers for all-cause mortality in the group with and without type 2 diabetes, respectively; Figure S4. The area under the curves for all-cause mortality in the group with and without type 2 diabetes in the KORA S4 and KORA-Age1 studies.

Additional file 2:  Table S1. Longitudinal association of single protein biomarkers with all-cause mortality among participants with and without type 2 diabetes in the KORA S4 sample (233 proteins); Table S2. Validation of results in KORA-Age1 for proteins significantly associated with all-cause mortality in the KORA S4 sample; Table S3. Associations of 35 validated proteins of all-cause mortality in the group with type 2 diabetes with cause-specific mortality in the pooled sample ( n  = 447); Table S4. Associations of 62 validated proteins of all-cause mortality in the group without type 2 diabetes with cause-specific mortality in the pooled sample ( n  = 2129); Table S5. 68 validated proteins for all-cause mortality and their association with cause-specific mortality and interaction effect with type 2 diabetes in the pooled sample; Table S6. Sensitivity analysis for all-cause and cause-specific mortality after exclusion of overlapping KORA S4 participants from the KORA-Age1 sample among participants with type 2 diabetes in the pooled sample ( n  = 402); Table S7. Sensitivity analysis for all-cause and cause-specific mortality after exclusion of overlapping KORA S4 participants from the KORA-Age1 sample among participants without type 2 diabetes in the pooled sample ( n  = 1943); Table S8. Sensitivity analysis considering other causes of death as competing risk among participants with type 2 diabetes in the pooled sample; Table S9. Sensitivity analysis considering other causes of death as competing risk among participants without type 2 diabetes in the pooled sample; Table S10. Proteins tested by other measurements in the KORA S4 sample; Table S11. Top pathways related to all-cause, cardiovascular, cancer-related and other-cause mortality enriched by their associated protein biomarkers; Table S12. Predictive performance for all-cause mortality after exclusion of overlapping KORA S4 participants from the KORA-Age1 sample; Table S13. Overlap of 68 mortality-related protein biomarkers identified in the present analysis with previous literature.

## Data Availability

The datasets analyzed in the current study are not publicly available due to German data protection laws, and restrictions were imposed by the ethics committee of the Bavarian Chamber of Physicians to ensure data privacy of the study participants but cooperation partners can obtain permission to use KORA data under the terms of a project agreement (https://helmholtz-muenchen.managed-otrs.com/external/).

## Abbreviations

BMI: Body mass index
cfNRI: Category-free net reclassification index
CHI3L1: Chitinase-3-like protein 1
C‐index: Concordance index
CVD: Cardiovascular disease
ELISA: Enzyme-linked immunosorbent assay
ECLIA: Electrochemiluminescence immunoassay
EN-RAGE: Protein S100-A12
FDR: False discovery rate
FGF-23: Fibroblast growth factor 23
GDF-15: Growth/differentiation factor 15
HDL: High-density lipoprotein
ICD: International Classification of Diseases
IDI: Integrated discrimination improvement
IGF: Insulin-like growth factor
IGFBP: Insulin-like growth factor-binding protein
IL: Interleukin
IL-1RA: Interleukin-1 receptor antagonist protein
IL-4RA: Interleukin-4 receptor subunit alpha
KIM1: Kidney injury molecule 1
KORA: Cooperative Health Research in the Region of Augsburg
Lasso: Least absolute shrinkage and selection operator
LIF-R: Leukemia inhibitory factor receptor
LOD: Limit of detection
LTβR: Lymphotoxin-beta receptor
MCP-3: Monocyte chemotactic protein 3
MERTK: Tyrosine-protein kinase Mer
MMP-12: Matrix metalloproteinase-12
MONICA: Monitoring of Trends and Determinants in Cardiovascular Disease
NT-proBNP: N-terminal prohormone brain natriuretic peptide
OGTT: Oral glucose tolerance test
PEA: Proximity extension assay
RARRES2: Retinoic acid receptor responder protein 2
SCORE: European Systematic COronary Risk Evaluation
T2D: Type 2 diabetes
TNFRSF: Tumor necrosis factor receptor superfamily member
TRAIL-R2: TNF-related apoptosis-inducing ligand receptor 2
