# Supplementary material for: Association of plasma proteomics with mortality in individuals with and without type 2 diabetes: Results from two population-based KORA cohort studies
Source: BMC Med. 2024 Sep 27;22:420. doi: 10.1186/s12916-024-03636-0 (PMC11438072; doi:10.1186/s12916-024-03636-0)
Supplement: Supplementary file 1 — Additional file 1: Figure S1. Inclusion and exclusion criteria for the present study; Figure S2. Kaplan–Meier curves for all-cause mortality, cardiovascular mortality, cancer-related mortality, and other-cause mortality stratified by baseline type 2 diabetes status in KORA S4 and KORA-Age1; Figure S3. Correlation between the validated 35 and 62 protein biomarkers for all-cause mortality in the group with and without type 2 diabetes, respectively; Figure S4. The area under the curves for all-cause mortality in the group with and without type 2 diabetes in the KORA S4 and KORA-Age1 studies. [file 12916_2024_3636_MOESM1_ESM.docx]

**Additional file 1**

**Association of plasma proteomics with mortality in individuals with and without type 2 diabetes: Results from two population-based KORA cohort studies**

Hong Luo, Agnese Petrera, Stefanie M. Hauck, Wolfgang Rathmann, Christian Herder, Christian Gieger, Annika Hoyer, Annette Peters, Barbara Thorand

**Contents**

[**Figure S1** Inclusion and exclusion criteria for the present study. 2](#_Toc163034833)

[**Figure S2** Kaplan-Meier curves for all-cause mortality, cardiovascular mortality, cancer-related mortality, and other-cause mortality stratified by baseline type 2 diabetes status in KORA S4 and KORA-Age1. 3](#_Toc163034834)

[**Figure S3** Correlation between the validated 35 and 62 protein biomarkers for all-cause mortality in the group with and without type 2 diabetes, respectively. 4](#_Toc163034835)

[**Figure S4** The area under the curves for all-cause mortality in the group with and without type 2 diabetes in the KORA S4 and KORA-Age1 studies. 5](#_Toc163034836)

**
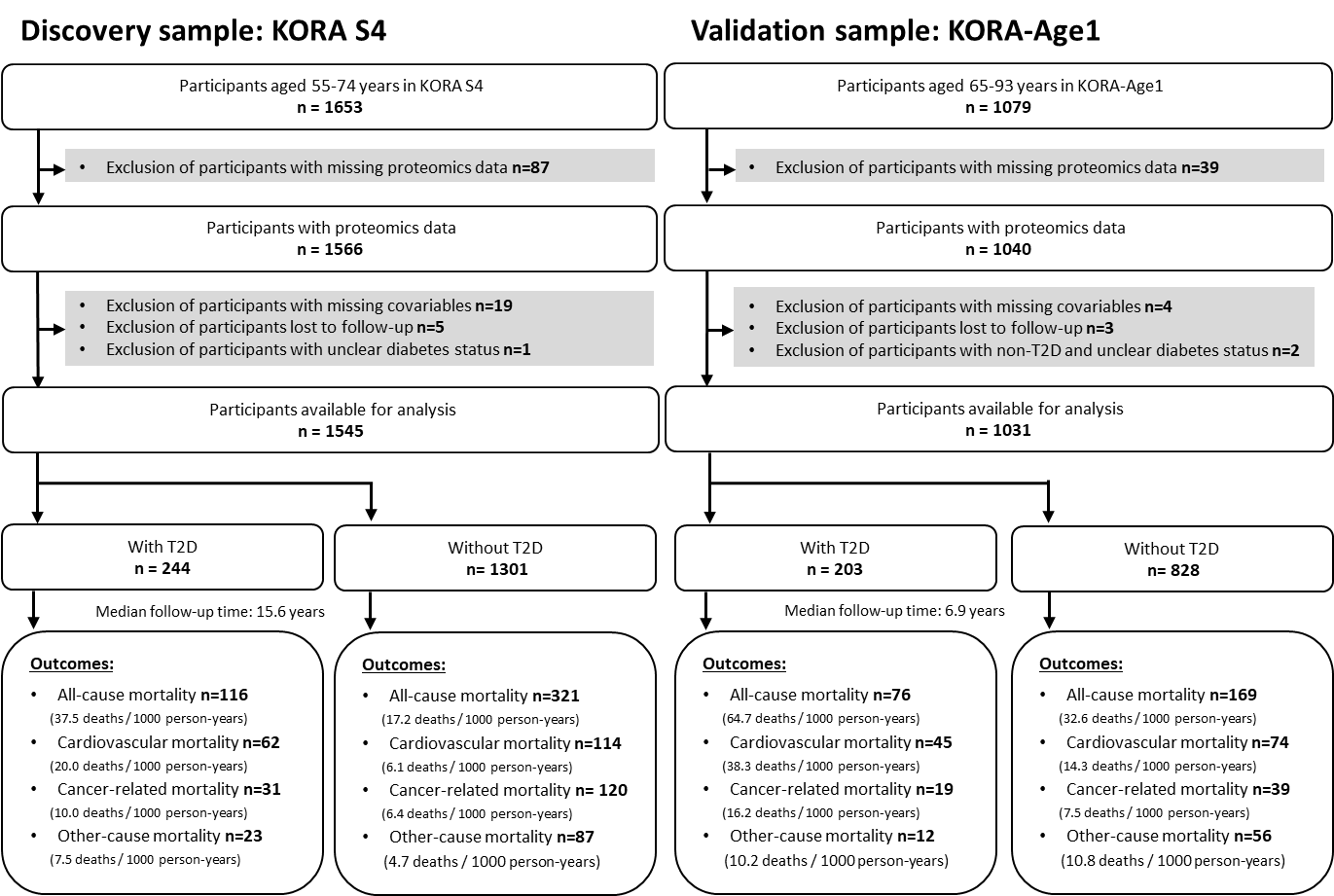
**

# **Figure S1** Inclusion and exclusion criteria for the present study. Abbreviations: KORA, Cooperative Health Research in the Region of Augsburg; T2D, type 2 diabetes.

**
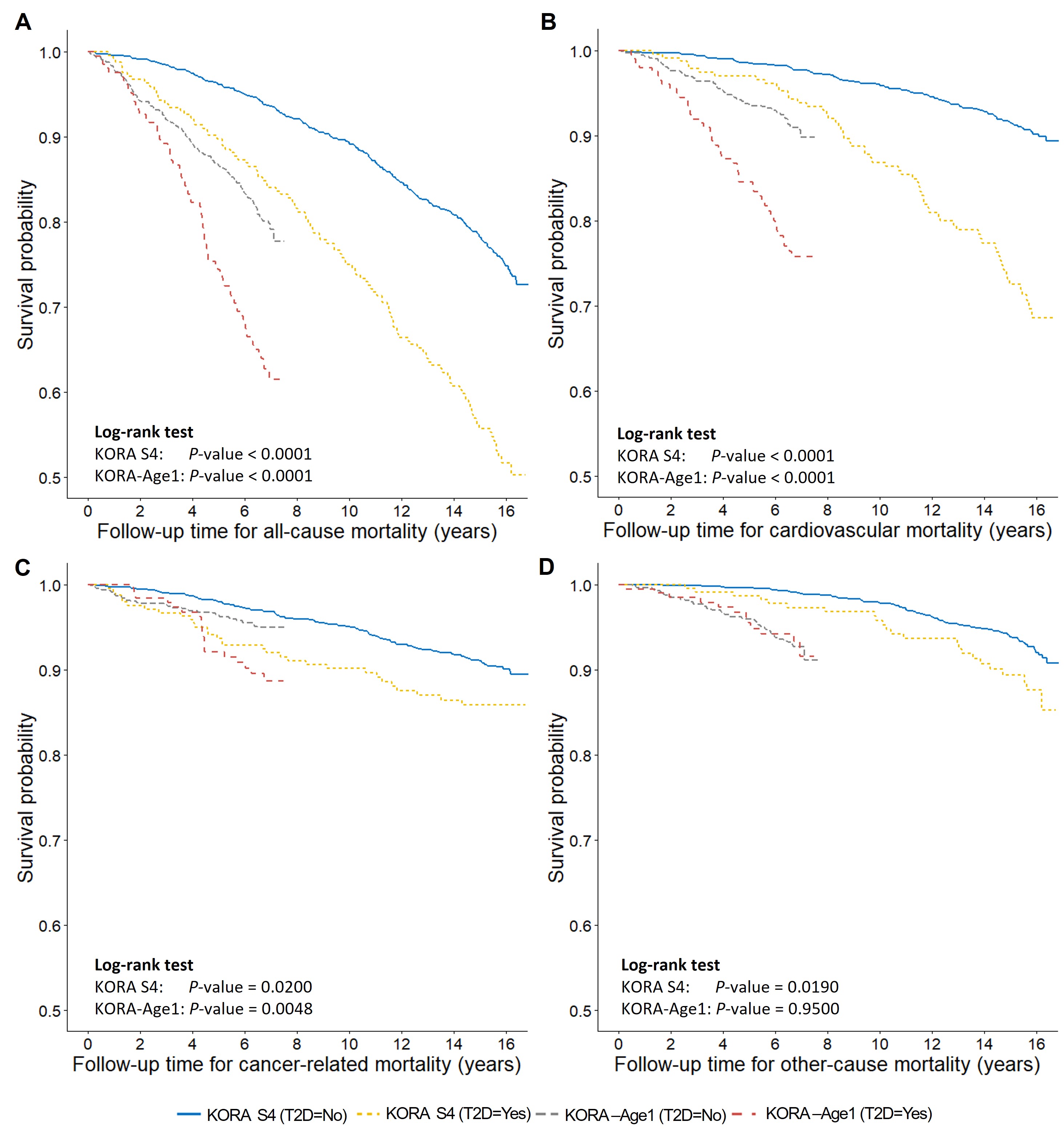
**

# **Figure S2** Kaplan-Meier curves for all-cause mortality, cardiovascular mortality, cancer-related mortality, and other-cause mortality stratified by baseline type 2 diabetes status in KORA S4 and KORA-Age1. (A) all-cause mortality; (B) cardiovascular mortality; (C) cancer-related mortality; and (D) other-cause mortality. The *P-*value < 0.05 for the log-rank test means statistically significant difference between the survival curves of the groups with and without baseline type 2 diabetes. Abbreviations: KORA, Cooperative Health Research in the Region of Augsburg; T2D, type 2 diabetes.


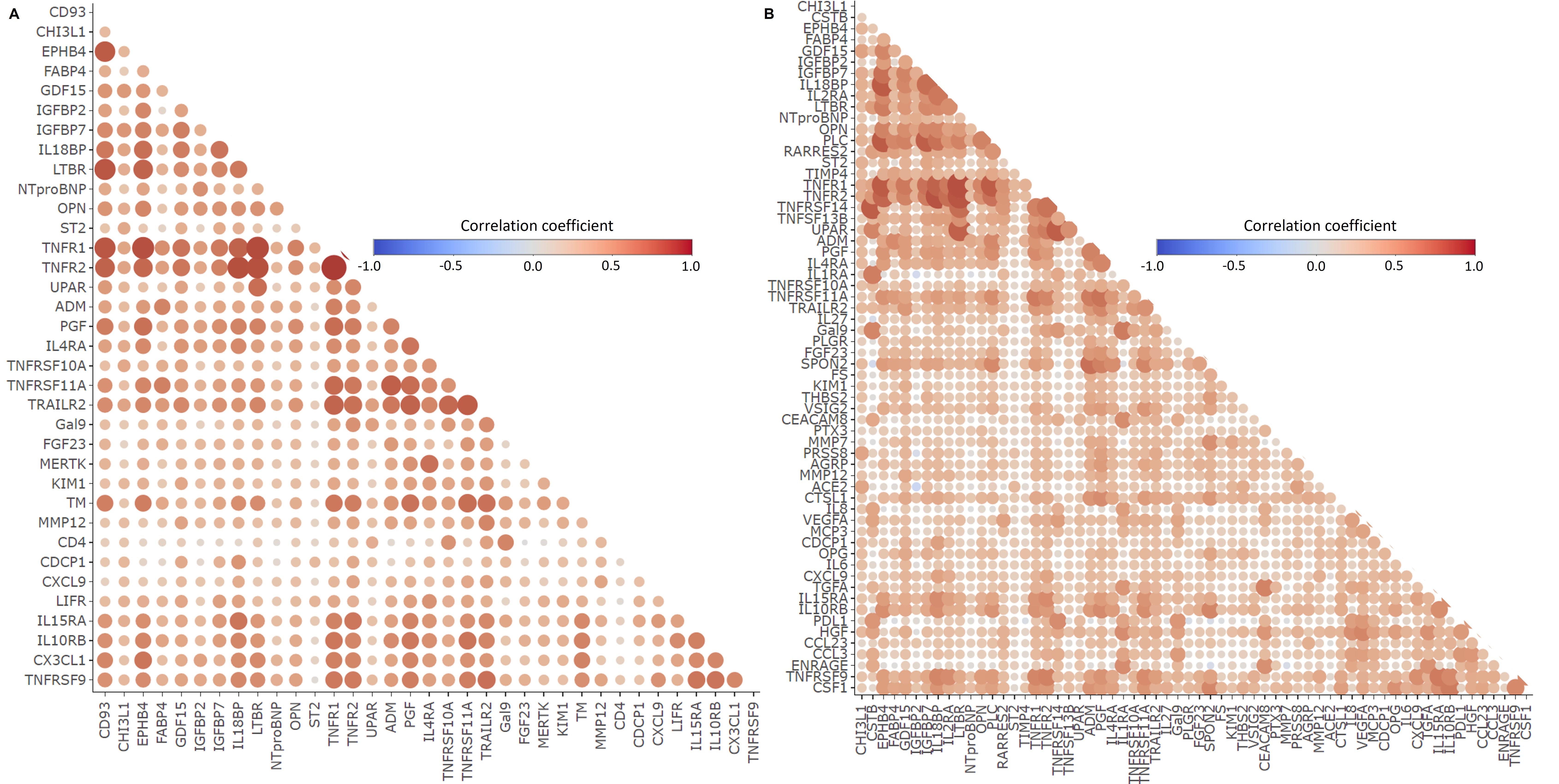


# **Figure S3** Correlation between the validated 35 and 62 protein biomarkers for all-cause mortality in the group with and without type 2 diabetes, respectively. (A) 35 protein biomarkers were identified in the group with type 2 diabetes; (B) 62 protein biomarkers were identified in the group without type 2 diabetes. The point size reflects the correlation test *P*-values. A bigger point size means a smaller *P-*value. Abbreviations: Full names of protein biomarkers can be found in Additional file 1: Table S1.


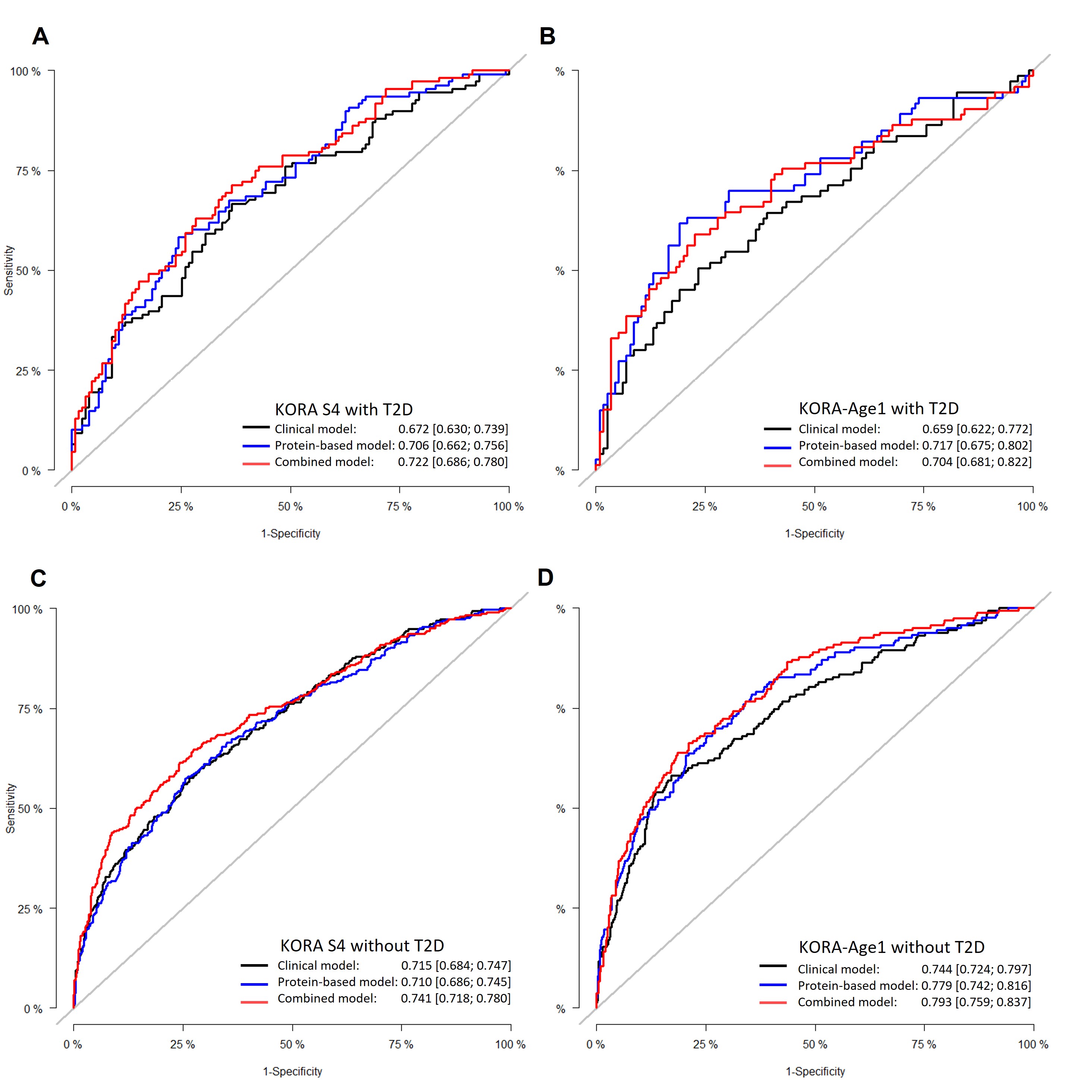


# **Figure S4** The area under the curves for all-cause mortality in the group with and without type 2 diabetes in the KORA S4 and KORA-Age1 studies. (A) group with T2D in the KORA S4 study; (B) group with T2D in the KORA-Age1 study; (C) group without T2D in the KORA S4 study; and (D) group without T2D in the KORA-Age1 study. Abbreviations: KORA, Cooperative Health Research in the Region of Augsburg; T2D, type 2 diabetes.
